# Segment Tracking via a Spatiotemporal Linking Process including Feedback Stabilization in an n-D Lattice Model

**DOI:** 10.3390/s91109355

**Published:** 2009-11-20

**Authors:** Babette Dellen, Eren Erdal Aksoy, Florentin Wörgötter

**Affiliations:** 1 Bernstein Center for Computational Neuroscience Göttingen, Max-Planck Institute for Dynamics and Self-Organization, Bunsenstrasse 10, 37073 Göttingen, Germany; 2 Institut de Robòtica i Informàtica Industrial (CSIC-UPC), Llorens i Artigas 4-6, 08028 Barcelona, Spain; 3 Bernstein Center for Computational Neuroscience Göttingen, Department for Computational Neuroscience, III. Physikalisches Institut, Georg-August University Göttingen - Biophysik, Friedrich-Hund Platz 1, 37077 Göttingen, Germany; E-Mails: eaksoye@bccn-goettingen.de (E.A.); worgott@bccn-goettingen.de (F.W.)

**Keywords:** model-free segment tracking, image motion, image segmentation

## Abstract

Model-free tracking is important for solving tasks such as moving-object tracking and action recognition in cases where no prior object knowledge is available. For this purpose, we extend the concept of spatially synchronous dynamics in spin-lattice models to the spatiotemporal domain to track segments within an image sequence. The method is related to synchronization processes in neural networks and based on superparamagnetic clustering of data. Spin interactions result in the formation of clusters of correlated spins, providing an automatic labeling of corresponding image regions. The algorithm obeys detailed balance. This is an important property as it allows for consistent spin-transfer across subsequent frames, which can be used for segment tracking. Therefore, in the tracking process the correct equilibrium will always be found, which is an important advance as compared with other more heuristic tracking procedures. In the case of long image sequences, *i.e.*, movies, the algorithm is augmented with a feedback mechanism, further stabilizing segment tracking.

## 1. Introduction

How can me make sense out of a complex visual scene having no or only little prior knowledge about its contents and the objects therein? Such problems occur, for example, if we wish to learn cause-effects in an hitherto unknown environment. Vice versa, many object definitions are only meaningful within the context of a given scenario and a set of possible actions.

Object tracking, *i.e.*, the assignment of consistent labels to objects in different frames of a video, is important for solving various tasks in the field of computer vision, including automatic surveillance, human-computer interaction and traffic monitoring [1]. Most object tracking algorithms require that predefined objects of interests are detected in the first frame or in every frame of the movie. However, in an unknown scenario, the tracking of image segments, presumably representing parts of objects, allows to postpone object definition to a later step of the visual-scene analysis. Several approaches for segment tracking have been proposed in the context of video segmentation [2–9]. Some approaches rely on segmenting each frame independently, e.g., by classifying pixels into regions based on similarity in the feature space, followed by a segment matching step based on their low-level features [2–5]. Other methods use motion projection to link segments, *i.e.*, the position of a segment in a future frame is estimated from its current position and motion features [6–9]. Cremers (2003) tracked motion segments by jointly solving motion segmentation and motion estimation by minimizing a single energy functional [10].

In the works described above, assumptions of the nature of objects being tracked or the data itself is being made either in the tracking procedure itself (matching assumptions) or already in the segmentation step, for example by assuming a priori a data model of some kind. In our work, we aim to reduce a priori assumptions on the data by choosing a data-driven method, *i.e.*, superparamagnetic clustering of data, for the segmentation procedure.

Superparamagnetic clustering finds the equilibrium states of a ferromagnetic Potts model defined by an energy function in the superparamagnetic phase [11–17]. The Potts model [11] is a generalization of the Ising model [18] which describes a system of interacting granular ferromagnets or spins, that can be in *q* different states, characterizing the pointing direction of the respective spin vectors. Depending on the temperature, *i.e.*, disorder introduced to the system, the spin system can be in the paramagnetic, the superparamagnetic, or the ferromagnetic phase. In the ferromagnetic phase, all spins are aligned, while in the paramagnetic phase the system is in a state of complete disorder. In the superparamagnetic phase regions of aligned spins coexists. Blatt *et al.* (1998) applied the Potts model to the image segmentation problems in a way that in the superparamagnetic phase regions of aligned spins correspond to a natural partition of the image data [15]. Finding the image partition corresponds to the computation of the equilibrium states of the Potts model. Importantly, the energy function used here only consists of interaction terms and does not contain a data term, hence no prior data model needs to be assumed, e.g. total number of segments or a priori defined pixel-label assignments. In this sense, the method is model free. In contrast, methods which find solutions by computing the minimum of an energy function usually require a data term—otherwise only trivial solutions are obtained. Hence, the equilibrium-state approach to the image segmentation problem has to be considered as fundamentally different from approaches finding the minimum energy configuration of energy functions in Markov random fields [19, 20]. As a consequence, the method is non-parametric and thus more generic, only requiring a pixel-pixel similarity criterium, e.g., gray value, color or texture, to be defined without making any further prior assumptions about the data.

The equilibrium states of the Potts model have been approximated in the past using the Metropolis-Hastings algorithm with annealing [21] and the methods on cluster updating, which are known to accelerate the equilibration of the system by shortening the correlation times between distant spins. Prominent algorithms are Swendsen-Wang [22], Wolff [23], and energy-based cluster updating (ECU) [16, 17]. In addition, the ECU algorithm has been shown to provide stable segmentation results over a larger temperature range (which is the main control parameter) than Swendsen-Wang [16, 17]. All of these methods obey detailed balance, ensuring convergence of the system to the equilibrium state. However, convergence has only been shown to be polynomial for special cases of the Potts model. The Potts model is known to be NP hard in the general case. The relaxation processes which can be emulated in spin-lattice models are often being used as approximations for synchronization processes within neural assemblies [12–17].

In our method, tracking of segments is accomplished through simultaneous segmentation of adjacent frames which are linked using local correspondence information, e.g., computed via standard algorithms for optic flow [24]. By performing motion projection and image segmentation within a single process we do not require any explicit matching of image segments, thus further reducing a priori assumptions that have to be made about the data. Synchronizing the segmentation process of adjacent frames has further the advantage that partitioning instabilities can be reduced. Usually, the segmentation (or partition) of an image is sensitive to global and local changes of the image, *i.e.*, small changes in illumination, the appearance/disappearance of objects parts, causing the partition to change from one frame to the next. To further stabilize segment tracking in the case of long image sequences, we developed a feedback control mechanism, which allows segmentation instabilities, e.g., sudden disappearances of segments, to be detected and removed by adjusting a control parameter of the segmentation algorithm.

Thus, the main contribution here is the development of a data-driven, model-free tracking algorithm, *i.e.*, no prior object knowledge is required in order to track image segments from frame to frame. Potential applications include moving object detection and tracking, and action recognition and classification from movies [25].

The paper is structured as follows: In Section 2, we extend the method of superparamagnetic clustering in spin models to the temporal dimension and introduce the controller algorithm. We also discuss the method in front of the background of energy minimization methods in Markov random fields. In Section 3, we first verify the core algorithm using short image sequences because these are more suitable to introduce and test the method. We further investigate the sensitivity of the algorithm to system parameters and noise. Then, we demonstrate that segment tracking can be achieved for real movies. The performance of the method is quantified in terms of partitioning consistency along an artificial image sequence. In Section 4, the results are discussed.

## 2. Algorithmic Framework

Segment tracking can be roughly divided into the following subtasks: (i) image segmentation, (ii) linking (tracking), and (iii) stabilization (tracking). The third point acknowledges that segments, unlike objects, are not per se stable entities, but are sensitive to changes in the visual scene. Subtasks (i–ii) will be solved using a conjoint spin-relaxation process emulated in an n-dimensional (n-D) lattice, which defines the core algorithm (Section 2.1). Local correspondence information for linking is obtained using standard algorithms for either stereo or optic flow [24, 26]. The conjoint segmentation approach has the advantage that the spin-relaxation processes of adjacent images synchronize, reducing partitioning instabilities.

Since simultaneous segmentation of long image sequences is practically impossible due to the high computational costs, we usually split the image sequence into a sequence of pairs. For example, the subsequent frames *t*_0_, *t*_1_ and *t*_2_ are split into two pairs {*t*_0_, *t*_1_} and {*t*_1_, *t*_2_}, where the last frame of previous pair is identical to the first frame of the next pair. If a segment of the last frame of {*t*_0_, *t*_1_} and a segment of the first frame of {*t*_1_, *t*_2_} occupy the same image region, we can assign the same segment label to both segments. This way segments can be tracked through the entire sequence. Since the algorithm preserves detailed balance (Section 2.1), spins can be transferred from one frame to the next, greatly reducing the number of iterations required to achieve a stable segmentation.

We further stabilize segment tracking by introducing a feedback controller (Section 2.2). In long image sequences, partitioning instabilities are likely to arise at some point during the tracking process. Thus, segments may be lost due to merging or splitting of segments. The feedback controller detects these kind of instabilities and adjusts a control parameter of the core algorithm to recover the original segments.

### 2.1. Core Algorithm

The method of superparamagnetic clustering has been previously used to segment single images [15–17]. Applying this framework to image sequences requires spin interactions to take place across frames. Due camera and object motion the images undergo changes during the course of time. To connect different frames, the mapping from one frame to the next needs to be known at least in some approximation. We solve this problem in the following way: Point correspondences, derived using algorithms for disparity or optic-flow computation, can be incorporated into the Potts model [11] by allowing spins belonging to different frames of the image sequence to interact if the respective pixels belong to locally corresponding image points. Then, spins belonging to the different frames of the sequences are relaxed simultaneously, resulting in a synchronized segmentation of the images of the sequence. The inter-frame spin interactions cause the spins of corresponding image regions to align, and, thus, they will be assigned to the same segment. Since the formation of segments is a collective process, the point correspondences do not have to be very accurate nor does the algorithm require point correspondences for each pixel. It is usually sufficient if the available correspondences capture the characteristics of the scene only roughly.

The aim of this work is to find corresponding image regions in image sequences, *i.e.*, stereo pairs and motion sequences. The segment tracking task is formulated as follows. Given an image sequence *S* containing points *p*(*x, y, t*) with coordinates (*x, y, t*) as elements, where *x* and *y* label the position within each image, while *t* labels the frame number, then we want to find a partitioning **P** = *P*_1_, .., *P_M_* of *S* in *M* groups such that

- *P_i_* ∩ *P_j_* = 0 and *P_i_* ≠ Ø for all groups
- if point *p* ∈ *P_i_*, then *s*(*p*, *P_i_*) *> s*(*p*, *P_j_*), where *s* is a function measuring the average distance of a point to the elements of a group and *i* ≠ *j*
- if *p*(*x_i_, y_i_, t_i_*) ∈ *P_r_*, then *p*(*x_i_* + △*x_i_, y_i_* + △*y_i_, t_i_* + 1) ∈ *P_r_*, where △*x_i_* and △*y_i_* are the shifts of point *p*(*x_i_*, *y_i_, t_i_*) along the *x* and *y* axes, respectively, from frame *t_i_* to frame *t_i_* + 1.

To perform this task, we assign a spin variable *σ_i_* (or label) to each pixel (or site) *i* of the image sequence. To incorporate constraints in the form of local correspondence information, we distinguish between neighbors within a single frame (2D bonds) and neighbors across frames (n-D bonds). We create a 2D bond (*i, k*)_2_*_D_* between two pixels within the same frame with coordinates (*x_i_, y_i_, t_i_*) and (*x_k_, y_k_,t_k_*) if

(1) $$\left|\left(x_{i}-x_{k}\right)\right|\;\leq \;{ɛ}_{2D}$$

(2) $$\left|\left(y_{i}-y_{k}\right)\right|\;\leq \;{ɛ}_{2D}$$

(3) $$t_{i}\;=\;t_{k}$$

where *ε*_2_*_D_* is the 2D-interaction range of the spins, a parameter of the system. Across frames, we create a n-D bond (*i, j*)*_nD_* between two spins *i* and *j* if

(4) $$\left|\left(x_{i}+d_{ij}^{x}-x_{j}\right)\right|\;\leq \;{ɛ}_{nD}$$

(5) $$\left|\left(y_{i}+d_{ij}^{y}-y_{j}\right)\right|\;\leq \;{ɛ}_{nD}$$

(6) $$t_{i}\;\neq \;t_{j}$$

(7) $$a_{ij}\;>\;\tau$$

where *ε_nD_* is the n-D interaction range. The values $d_{ij}^{x}$ and $d_{ij}^{y}$ are the shifts of the pixels between frames *t_i_* and *t_j_* along the axis *x* and *y*, respectively, obtained from the optic-flow map or disparity map. The parameters *a_ij_* ∈ [0, 1] are the respective amplitudes (or confidences) of the optic-flow or stereo algorithm. A large amplitude suggests a large confidence in the computed local correspondence. Parameter *τ* is a threshold, removing all local correspondences having a small amplitude.

We define for every bond on the lattice the distance

(8) $$\Delta_{\text{ij}}=\left|g_{i}-g_{j}\right|$$

where *g_i_* and g*_j_* are the feature vectors, e.g., gray value, color, or texture, of the pixels *i* and *j*, respectively. The mean distance △̄ is obtained by averaging over all bonds. We further define an interaction strength

(9) $$J_{ij}=1-\Delta_{ij}/\bar{\Delta }$$

The spin model is now implemented such a way that neighboring spins with similar color have the tendency to align. We use a *q*-state Potts model [11] with the Hamiltonian

(10) $$H=-\sum_{{\langle ik\rangle }_{2D}}J_{ik}\delta (\sigma_{i}-\sigma_{k})-\sum_{{\langle ij\rangle }_{nD}}J_{ij}\delta (\sigma_{i}-\sigma_{j})$$

Here, 〈*ik*〉_2_*_D_* and 〈*ij*〉*_nD_* denote that *i, k* and *i, j* are connected by bonds (*i, k*)_2_*_D_* and (*i, j*)*_nD_*, respectively The Kronecker *δ* function is defined as *δ*(*a*) *=* 1 if *a* = 0 and zero if otherwise. The segmentation problem is then solved by finding clusters of correlated spins in the low temperature equilibrium states of the Hamiltonian *H*. The temperature parameter determines the amount of disorder introduced to the system. The spin states have to be observed over several iterations to identify clusters as groups of correlated spins. The total number *M* of segments is then determined by counting the computed segments. The spin states *σ_i_* can take integer values between 1 and *q*, where *q* is a parameter of the algorithm. The number of segments is not constrained by the parameter *q*. Please note that segment labels are not identical to spin states. Spins which belong to the same segment are always in the same spin state, however, the reverse is not necessarily true. Note that the local correspondences used in the algorithm to create n-D bonds are precomputed and are not altered or optimized when computing the equilibrium state. The computation of local correspondences is not the aim of this paper.

**Figure 1.**
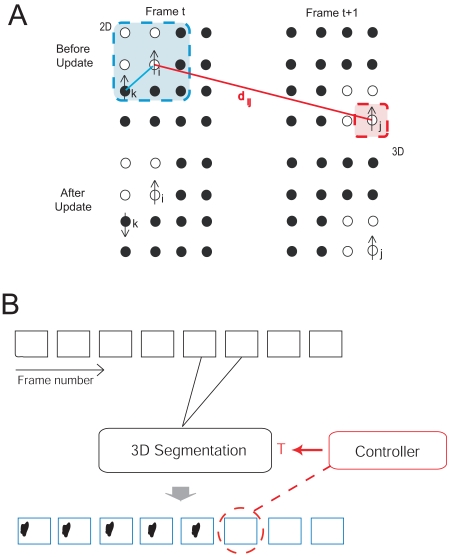
**A** The spin states (upward and downward pointing arrows) of pixels *i, k*, and *j* are shown before and after a spin update for two adjacent frames *t* and *t*+1 of an image sequence. The white and black circles indicate pixels of small and large gray values, respectively. Pixel *i* interacts with pixels *k* and *j* in its 2D and 3D neighborhood (shaded areas), respectively, which are in the same spin state. **B** Pairwise (3D) segmentation of movies. A feedback controller detects segmentation instabilities and adjusts the control parameter *T* of the core algorithm (3D segmentation) to recover lost segments.

We find the equilibrium spin configuration using a clustering algorithm. In a first step, “satisfied” bonds, *i.e.*, bonds connecting spins having identical spin values *σ_i_* = *σ_j_*, are identified. Then, in a second step, the satisfied bonds are “frozen” with a some probability *P_ij_*. Pixels connected by frozen bonds define a cluster, which are updated by assigning to all spins inside the same clusters the same new value [22]. In the method of superparamagnetic clustering proposed by Blatt *et al.* (1996) [15] this is done independently for each cluster. In this paper, we will employ the method of energy-based cluster updating (ECU), where new values are assigned in consideration of the energy gain calculated for a neighborhood of the regarded cluster [16, 17]. A schematic of the spin system of an image sequence is depicted in Figure 1A.

The ECU algorithm computing the equilibrium of *H* consists of the following steps:

1. Initialization: A spin value *σ_i_* between 1 and *q* is assigned randomly to each spin *i*. Each spin represents a pixel of the image sequence.
2. Computing bond freezing probabilities: If two spins *i* and *j* are connected by a bond and are in the same spin state *σ_i_* = *σ_j_*, then the bond is frozen with a probability
  (11) $$P_{ij}=1-\exp(-0.5J_{ij}/T)$$
  Negative probabilities are set to zero.
3. Cluster identification: Pixels which are connected by frozen bonds define a cluster. A pixel belonging to a cluster *u* has by definition no frozen bond to a pixel belonging to a different cluster *v*.
4. Cluster updating: We perform a Metropolis update [22, 27] that updates all spins of each cluster simultaneously to a common new spin value. The new spin value for a cluster *c* is computed considering the energy gain obtained from a cluster update to a new spin value *w_k_*, where the index *k* denotes the possible spin values between 0 and *q*, respectively Note that this new spin value is, once chosen, a constant and has to be distinguished from the spin variables *σ_i_*. Updating the respective cluster to the new value results in a new spin configuration $W_{k}^{c}$.
  The probability for the choosing the new spin value *w_k_* for cluster c is computed by considering the interactions of all spins in the cluster *c* with those outside the cluster, assuming that all spins of the cluster are updated to the new spin value *w_k_*, giving an energy
  (12) $$E(W_{k}^{c})=\sum_{i\in c}\left[-\sum_{\begin{array}{l} {\langle ij\rangle }_{2D}\\ c_{k}\neq c_{j} \end{array}}\eta J_{ij}\delta (\sigma_{i}-\sigma_{j})-\sum_{\begin{array}{l} {\langle ij\rangle }_{nD}\\ c_{k}\neq c_{j} \end{array}}\eta a_{ij}J_{ij}\delta (\sigma_{i}-\sigma_{j})\right]$$
  where 〈*ik*〉_2_*_D_*, *c_k_* ≠ *c_j_* and 〈*ij*〉*_nD_*, *c_k_* ≠ *c_j_* are the noncluster neighborhoods of spin *i*. Here, *N* is the total number of pixels of the image sequence. The constant *η* is chosen to be 0.5.
  Similar to a Gibbs sampler, the selecting probability $P(W_{k}^{c})$ for choosing the new spin value to be *w_k_* is given by
  (13) $$P(W_{k}^{c})=\exp(E(W_{k}^{c}))/\sum_{l=1}^{q}\exp(E(W_{l}^{c}))$$
5. Iteration: The new spin states are returned to step 2 of the algorithm, and steps 2–5 are repeated, until the total number of clusters stabilizes, *i.e.*, the change of the number of clusters is small compared to the change of the number clusters during the first ten iterations (see also Figure 5F). In practice, we usually use a fixed number of iterations.
6. Segments are defined as groups of correlated spins and can be extracted using a thresholding procedure. All pairs of pixels connected by a bond (*i, j*) with *c*(*σ_i_*, *σ_j_*) > *θ* are considered to be friends, *i.e.*, they must belong to the same segment. The function *c* computes the correlation of the spin states of *i* and *j* over several iterations. Then, all mutual friends are assigned to the same segment. Finally, *M* is determined by counting the total number of segments. In practice, we find it sufficient to take the clusters found in the last iteration as segments.

In an earlier study we had provided evidence that this algorithm obeys *detailed balance*. The full proof shall not be repeated here and can be found in [16]. Detailed balance assures that the proposed algorithm computes an equilibrium spin configuration of the energy function and that this is Boltzmann distributed. However, the equilibrium might not be found in polynomial time (see Section 2.3).

The consequence of detailed balance is that spin states can be transferred across image pairs, where spins are being calculated for one pair (the first pair) and then pixels in the next two frames (the second pair) are just assigned these spins from where on a new relaxation process starts (see Figure 5 for an example). Hence, the relaxation process for the second pair (and all to follow) is much faster when using spin transfer and the system will always arrive at the thermodynamic equilibrium making spin-transfer based segmentation concordant across frames. Note, this property allows consistent segment tracking across many frames without additional assumptions (see Figure 5), which requires more effort with other methods.

The following should be noted. In this method, bonds between adjacent frames are created from the precomputed optic-flow or disparity maps and frozen with a probability that depends on the feature similarity of the respective corresponding pixels. Whether these bonds are frozen in the final configuration (*i.e.*, the respective proposed point correspondences are accepted), and thus promote the formation of a certain segment correspondence, is decided inherently by finding the equilibrium state of Equation 10. This procedure makes the method more robust against errors in the optic-flow or disparity map.

### 2.2. Feedback Control

Segmentation instabilities arising during the tracking process can be partly removed by adjusting the temperature parameter of the core algorithm. The temperature choice affects the formation of segments, hence, a segment which has been lost in a previous frame can sometimes be recovered by increasing the temperature for a certain period.

The feedback controller tracks the size of the segments and reacts if the size of a segment changes suddenly. The first controller function

(14) $$P_{C}^{j}(t)=\{\begin{array}{ll} 1 & \text{if}\;\Delta S_{j}(t)<\tau_{1},\\ \exp[-\Delta S_{j}(t)/\alpha ]/\beta & \text{othewise}, \end{array}$$

measures the probability of change of segment *j*, where *S_j_*(*t*) is the size of segment *j* at frame *t* and △*S_j_*(*t*) = *S_j_*(*t*) − *S_j_*(*t* − 1), and *α, β, τ*_1_ are constant parameters. The history of segment *j* in terms of occurrence is captured by the second controller function

(15) $$P_{H}^{j}(t)=0.4H_{j}(t-1)+0.3H_{j}(t-2)+0.2H_{j}(t-3)+0.1H_{j}(t-4)$$

with *H_j_*(*t*) = 1 if *S_j_*(*t*) > 0 and zero otherwise.

Segmentation instabilities may cause a segment to be lost, for example through segment merging or splitting. We define two threshold parameters *τ*_2_ and *τ*_3_. An unexpected segment loss is detected by the controller if the conditions

(16) $$S_{j}\;=\;0$$

(17) $$P_{C}\;<\;\tau_{2}$$

(18) $$\text{and}\;P_{H}\;>\;\tau_{3}$$

are fulfilled. An unexpected segment appearance is detected by the controller if the conditions

(19) $$P_{C}\;<\;\tau_{2}$$

(20) $$\text{and}\;P_{H}\;<\;\tau_{3}$$

are fulfilled. The identities of the affected segments are stored by the controller. The temperature of the core algorithm is varied using predefined temperature steps △*T*. The segmentation is repeated at the new temperature *T* + △*T* only for the affected segments of the frames. If the lost segments can be recovered at one of these temperatures, the affected segments are relabeled accordingly. Furthermore, the new temperature *T* + △*T* values of the affected segments are inherited to the next frame in order to prevent the same segmentation error. Note that the temperature value can be increased to a predefined maximum value, otherwise undesired artifacts might be observed.

A schematic of the entire system, *i.e.*, core algorithm with feedback control, is presented in Figure 1B.

### 2.3. Comparison with Energy-Minimization-Based Methods in Markov Random Fields

The method described in this paper is compared with energy minimization in Markov random fields. Similarities and differences of the approaches are analyzed and summarized in this section. Particular attention is given to combinatorial graph cuts methods, which have provided powerful computer vision algorithms for stereo, motion, image restoration, and image segmentation in recent years [19, 20].

The image segmentation problem has been previously formulated in terms of finding a discrete labeling *f* that minimizes the Potts energy function

(21) $$E(f)=E_{\text{smooth}}(f)+E_{\text{data}}(f)$$

where the data term

(22) $$E_{\text{data}}(f)=\sum_{p\in P}D_{p}(f_{p})$$

measures how well label *f_p_* fits the pixel *p* given the observed data, and the interaction term

(23) $$E_{\text{smooth}}(f)=\sum_{(p,q)\in P}J_{pq}T(f_{p}\neq f_{q})$$

where *T*(·) is one if the statement inside is true and zero otherwise [19]. Note that the formulation of the Potts interaction energy is slightly different from the standard ferromagnetic case used by us. The energies here are positive and *T*(·) is zero if two pixels have the same labels. The data term has to be included in the energy function, because otherwise the energy minimum would be found at a configuration where all pixels have the same label, and thus *E*_smooth_ = 0. We are assuming *J_p,q_* to be positive for all *p, q* (otherwise the system would describe a spin glass or an antiferromagnet). Hence, some knowledge about the solution needs to be provided when defining the data term, and as such the method is *model driven*. This constitutes a major difference to the method used in this paper. Superparamagnetic clustering requires no data term, *i.e.*, no knowledge about the solution needs to be provided in the beginning, expect from a pixel-pixel similarity criterium.

Various techniques have been proposed to find the minimum energy configuration, e.g., simulated annealing [21], belief propagation [28], the Swendsen-Wang-based method of Barbu and Zhu [22, 29] and graph cuts [20]. Combinatorial graph-cut techniques have received a lot of attention in recent years because they provide polynomial-time algorithms for the two-label problem [20]. However, finding the minimum of the energy function of Equation 21 is known to be NP hard for more than two labels [19, 20]. For the multi-label problem graph cuts can be used iteratively [19]. In the *α*/*β* swap algorithm groups of pixels are assigned a new label simultaneously, while the new best move is computed used using graph cuts while considering only two labels *α* and *β* at a time.

The method of superparamagnetic clustering also formulates an energy function (see Equation 10) describing a Potts model, which is a specific example of a Markov Random Field. Here, *no* energy minimum is computed. Instead, the equilibrium state at a given temperature (in the superparamagnetic regime) is approximated using similar techniques as used for energy miminization, e.g., simulated annealing [21] and cluster-update algorithms, such as Swendsen-Wang [22], Wolff [30], and ECU [16, 17]. Note that combinatorial graph cuts is not applicable here. Our model does not contain a data term, which would be required to define the connections of the graph nodes to the two special nodes called the source and the sink of min-cut/max-flow algorithms. If simulated annealing is employed, one starts at a high temperature and slowly reduces the temperature until one reaches the target temperature. Cluster-update algorithms however do not require annealing to approximate the equilibrium spin configuration and are much more efficient than Metropolis simulations using single moves only [16, 17]. Similar to the *α/β* swap algorithm and the algorithm of Barbu and Zhu [19, 20, 29], the ECU algorithm moves groups of pixels simultaneously to reach faster convergence and also selects “best moves”.

Finding the equilibrium states in the Potts model is in general NP hard. In practice, the Swendsen-Wang algorithm often shows fast convergence to the equilibrium state, unlike the Metropolis-Hastings algorithm, which usually requires exponential time. For certain instances of the Potts model, rapid mixing, *i.e.*, polynomial-time convergence to the equilibrium state, has been proven for the Swendsen-Wang process [31]. In the general case, however, Swendsen-Wang shows exponential mixing [32]. It remains an open question whether ECU mixes rapidly for special cases of the Potts models.

For the 2D Ising model without an external field, *i.e.*, two-label Potts model, polynomial-time algorithms for computing the partition function exist. Nobel laureate Lars Onsager was the first who obtained a closed-form solution of the partition function for the 2D Ising model on a square lattice [33]. However, three-dimensional models and the 2D case with external field are also assumed to be NP hard.

## 3. Results

We apply the algorithm to various synthetic and real image sequences. Unless otherwise indicated, the following parameter values *q* = 10, *ε*_2_*_D_* = 1, and *ε_nD_* = 0.5 are used. In Section 3.1, the core algorithm is applied to short image sequences and a sensitivity analysis is performed. Temperatures in the range of *T* = 0.05 – 0.3 are chosen for segment tracking, as suggested by the sensitivity analysis. Then, in Section 3.2, feedback control is added and the algorithm is applied to movies.

### 3.1. Verification of Core Algorithm

We first use stereo image pairs and a three-frame motion sequence to test and verify the core method (Section 2.1) before applying the algorithm to long image sequences, *i.e.*, movies. Stereo images are more suitable to illustrate the basic properties of the algorithm. In the following figures we use spin states instead of cluster labels to limit the total number of colors in the color-coded images to a maximum value of *q*. Please note that the spin states are not identical to the cluster labels (see also Section 2.1).

#### Illustrating Example: Artificial Solid Square

**Figure 2.**
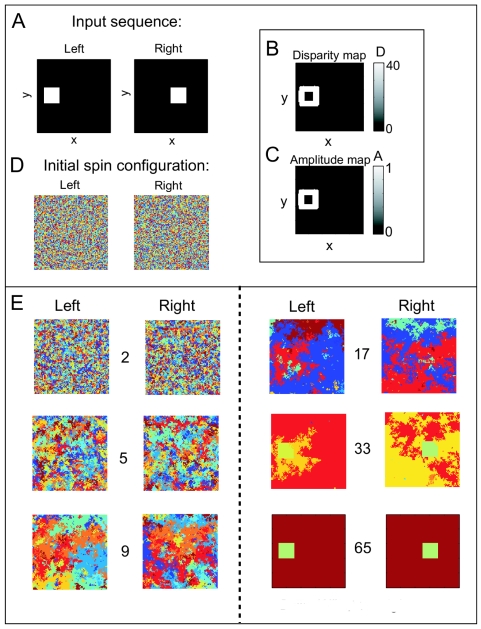
Solid-square stereo pair. **A** The input image consists of a square which is shifted by a disparity value of 40 pixels from one frame to the other, labeled left and right. **B** By applying a stereo algorithm, the disparity map *D* of the stereo pair could be computed, returning reliable disparity values at the edges. **C** The corresponding amplitude map *A* reflecting the confidence in the computed stereo values is shown. **D** The spin states are initialized randomly to values between 1 and 10. **E** The spin states after 2, 5, 9, 17, 33, and 65 iterations are shown. Progressive agglomeration of clusters of aligned spins can be observed until a stable configuration is reached.

We first demonstrate the algorithm for a synthetic scene which contains a single, solid square, which is shifted by a disparity value of 40 pixels along the *x* axis, resulting in an image sequence containing two frames, labeled left and right (see Figure 2A, left and right panel). Each image is of size 100 × 100 pixels^2^. We estimate the disparity of the pixels by applying a stereo algorithm [26], which returns a disparity map *D* and an amplitude map *A*, shown in Figure 2B,C, respectively. The disparities and the respective amplitudes determine whether a pixel in the right frame is a neighbor of a pixel in the left frame. Clustering is performed with *T* = 0.01. The spin states of the spin system are initialized to randomly chosen discrete values between 1 and 10, as depicted in Figure 2D, left and right panel. Then, the system is evolved using the energy-based cluster update algorithm described in the previous section. The spin states after 2, 5, 9, 17, 33, 65 iterations of the algorithm are shown in Figure 2E. The process of cluster formation can be easily followed through the iterations. At iteration step 65, the pixels belonging to the square in both the left and the right frame have been assigned to the same cluster, despite incomplete disparity information.

#### Sensitivity Analysis

**Figure 3.**
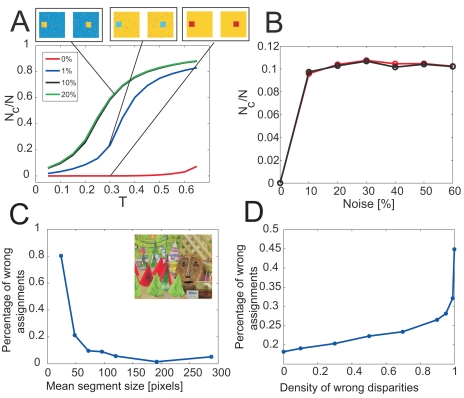
Sensitivity analysis. **A** The ratio of the total number of clusters *N_c_* divided by the total number of pixels *N* is plotted as a function of the parameter *T* for different realizations of Gaussian noise, having standard deviations from the absolute gray-value difference of object and background of 0%, 1%, 10%, and 20%. Here, ground truth disparity was used. The segmentation results are shown for *T* = 0.3 and different noise levels. **B** The ratio *N_c_/N* is plotted as function of the noise level using the ground-truth disparity map (red line) and the disparity computed with a phase-based stereo algorithm [26] (black line). **C** Percentage of wrongly assigned image points as a function of the mean segment length for the Cones stereo pair (left image see inset). Here the ground truth disparity map was used. **D** Total percentage of wrongly assigned image points as a function of the density of erroneous disparities. Here, the ground truth disparity was corrupted with noise to obtain an erroneous mapping.

We investigate the sensitivity of the algorithm with regard to the parameter *T* for different levels of Gaussian white noise that we add to the solid-square stereo pair. In Figure 3A, the ratio of the averaged number of clusters after 100 iterations, computed from 10 runs of the algorithm, and the total number of pixels is plotted as a function of the temperature *T* for four different realizations of Gaussian noise with standard deviations from the absolute gray-value difference of object and background of 0%, 1%, 10%, and 20%, depicted in red, blue, black, and green, respectively. For this sequence, a perfect segmentation is achieved for *N_c_* = 2, corresponding to *N_c_/N* = 10^−4^. For a noise level of 0%, the performance of the algorithm is only weakly sensitive to changes in temperature (red line). However, when adding noise to the images, the algorithm becomes more sensitive to changes in temperature (blue line), but fast saturates for increasing noise levels (black and green line). For each noise level, the segmentation results are depicted for *T* = 0.3. To establish the 3D neighborhood of image pixels here, the ground-truth disparity map of the image pair was used. However, usually, when adding noise, the quality of the disparity map decreases. Consequently, we also investigated the performance of the algorithm when computing the disparity map with a phase-based stereo algorithm. In Figure 3B, the ratio of the number of clusters and the total number of image pixels is depicted as a function of the noise level at temperature *T* = 0.1 (black line). The ratios when using the ground truth disparity is plotted for comparison (black line). In this example, the performance is independent of the quality of the disparity map.

We further investigate the performance of the algorithm with respect to establishing correspondences on the example of the Cones stereo pair (http://vision.middlebury.edu/stereo/). The left frame of the Cones stereo pair is shown as an inset of Figure 3C. The percentage of wrongly assigned image points was computed independently for every segment, and the average percentage of wrongly assigned image points was plotted as a function of the mean length of the segments (Figure 3C). A segment of length *l* contains *l*^2^ image points. The plot demonstrates that the performance of the algorithm is higher for large segments than for small segments, confirming our expectation that color segmentation works best for large uniform image regions. In textured areas, corresponding to very small segment sizes, the performance of the algorithm decreases rapidly.

We also investigated the influence of errors in the precomputed disparity on the performance of the algorithm by replacing disparity values of the ground-truth map randomly by erroneous values ranging from 0 to *n*, where *n* is the width of the image. In Figure 3D the total percentage of wrongly assigned image points (taken from all segments) is plotted as a function of the density of erroneous disparity values. As expected, the performance decreases with increasing error in the disparity map. In summary: one finds that the errors are in general small and the error curves flat for larger segments corresponding to non-textured regions. It is evident that all gray (color) difference based segmentation algorithms in general do not capture textured regions and the increasing errors for small segments reflect this situation. On the other hand, it is very assuring that those segments, which follow from larger consistent gray (color) value similarities, are indeed only little affected by errors in the (stereo-)correspondence map.

#### Real Stereo Pair

**Figure 4.**
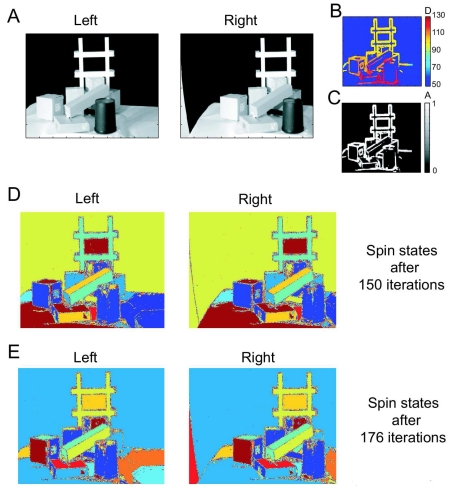
Cluttered-objects stereo pair. **A** Stereo image pair showing a cluttered scene containing a variety of objects. **B-C** The dense stereo algorithm returns mainly disparity information the edges of the objects. **D-E** The spin states computed by the clustering algorithm are shown after both 150 and 176 iterations for easier visual identification of the segments.

This stereo pair shows two views of a scene of cluttered objects, *i.e.*, paper boxes, a trash can, and a white Styrofoam object (Figure 4A, left and right panel). Each image is of size 180 × 380 pixels^2^. This stereo pair is demanding because of the amount of occlusion, the light reflexions, shadows, and the large disparities, which lead to perspective distortions, posing a problem to approaches based on segment matching. The stereo algorithm returns reliable disparity values at the edges (Figure 4B,C). Otherwise, the amplitude is zero (Figure 4C). However, when performing clustering with *T* = 0.2, the algorithm is still able to segment most of the boxes into their composite surfaces (Figure 4D,E). Some of the surfaces are partly shattered though, due to light reflexions and shadows, breaking the uniformity of the surfaces. Both the spin states after 150 and 176 iterations are shown to allow easier identification of correlated spins through visual inspection.

#### Real Motion Sequence

So far we had been validating our method using synthetic and real stereo pairs. Now we demonstrate that spatiotemporal synchronization of spins enables segments to be tracked through the frames of real movies.

**Figure 5.**
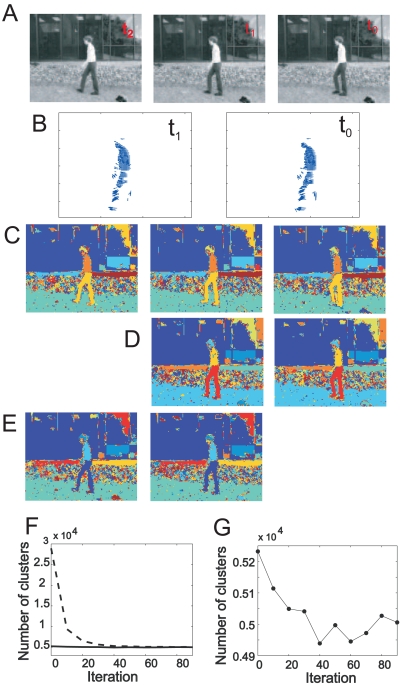
**A** Three frames of a walking sequence, labeled *t*_0_, *t*_1_, and *t*_2_, respectively. **B** Optic-flow vectors coding the mapping from frame *t*_0_ to *t*_1_, and *t*_1_ to *t*_2_. **C** Spin states after 100 iterations. **D** Spin states for a sequence consisting of frames *t*_0_ and *t*_1_. **E** Spin states for a sequence consisting of *t*_1_ and *t*_2_. **F** The number of clusters as a function of the iteration number for the first sequence containing frame *t*_0_ and *t*_1_ (dashed line) and for the second sequence containing frame *t*_1_ and *t*_2_ (solid line). **G** Enlarged plot of the second sequence.

We apply the core algorithm to three frames of a motion sequence showing a woman walking from the right to the left. The sequence was obtained from http://www.cs.brown.edu/black/∼ images.html. The frames are of sizes 118 × 158 pixels^2^ (Figure 5A). To compute optic flow, any standard algorithm can be used [24], e.g., a differential technique by Lucas and Kanade [34]. Here we used a method proposed in [35]. The performance of the segmentation is only weakly sensitive to the quality of the optic-flow estimation. The optic-flow fields, coding the mapping from the frame *t*_0_ to frame *t*_1_, and from frame *t*_1_ to frame *t*_2_, are depicted in Figure 5B. The spin states after 100 iterations are shown in Figure 5C. The algorithm successfully segmented the legs, the arms, a part of the head, and parts of the background, which thus can be tracked from frame to frame. For the highly textured area in the background, no stable 3D clusters could form since the gray-value similarity of neighboring pixels is too low. However, texture could be treated by performing segmentation based on texture similarity instead of color similarity.

When analyzing long motion sequences, it is inefficient to apply the algorithm to all frames at once because the computational costs increase with the number of pixels. Hence, we split the sequences in pairs of two frames at a time, where the last frame of the previous sequence is identical with the first frame of the next sequence. Then, we initialize the spin states of each sequence with the final spin states of the previous sequence. The spin states for the first sequence containing frame *t_0_* and *t*_1_ after 100 iterations are shown in Figure 5D. Then, the algorithm is applied to the next pair, containing frame *t*_1_ and *t*_2_, where the spin states of both frame have been initialized to the final spin states of frame *t*_1_ of the previous sequence. The spin states after 13 iterations are shown in Figure 5E, demonstrating that the number of iterations required to achieve a satisfying segmentation result is greatly reduced by this technique. The number of clusters for the first sequence and the second sequence are displayed as a function of the iteration number in Figure 5F, dashed and solid line, respectively. The number of clusters for the second sequence is plotted as a function of the iteration number at a different scale (Figure 5G). Initially, the number of clusters decreases slightly and then approaches a stable state. In a motion sequence, the number of clusters is expected not to change much from one frame to the next. Mainly the boundaries of the clusters reorganize during the first iterations.

The segments of adjacent image pairs are connected as follows. Two segments belonging to the segmentation of frame *t*_1_ of pair {*t*_0_, *t*_1_} and frame *t*_1_ of pair {*t*_1_, *t*_2_}, respectively, are assigned the same label if they occupy the same region in image frame *t*_1_. For this purpose, we compute the percentage of the cluster areas which overlap in the image space. If the overlap is larger than a fixed threshold, the clusters are assigned the same label. This way we can track the segment through the whole sequence.

### 3.2. Segment Tracking with Feedback Stabilization

**Figure 6.**
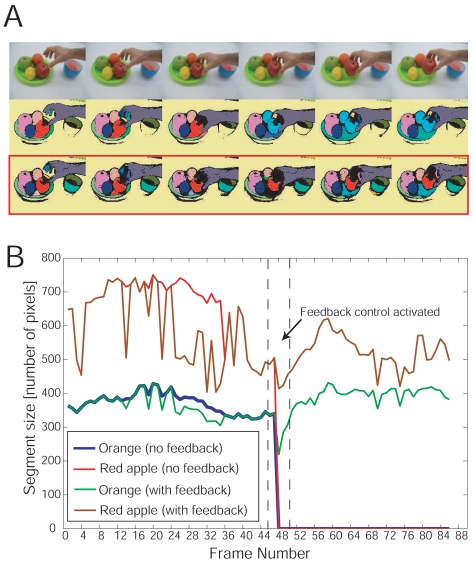
Feedback control for segmentation stabilization. **A** A few frames of a movie showing a hand taking a red apple from a plate are shown together with the results of the core algorithm without and with feedback control (upper, middle, and lower panel, respectively). **B** The segment size is plotted as a function of the frame number for the segments representing the red apple and the orange without and with feedback control, depicted as red, blue, brown and green lines, respectively. At frame number 45 the segment sizes of the red apple and the orange drop unexpectedly to zero (red and blue lines), and the feedback control is activated, increasing the temperature *T* until the original segments are recovered (brown and green lines).

We add feedback control (see Section 2.2) with parameters *α =* 200 pixels, *β =* 0.8, *τ*_1_*=* 50 pixels, *τ*_2_ = 0.9, and *τ*_3_ = 0.6 to the core algorithm with temperature *T* = 0.05 and apply the algorithm to long image sequences. The first movie shows a hand taking a red apple from a plate with several fruits. A few frames of the movie are depicted in the upper panel of Figure 6A. If the core algorithm is applied at constant temperature without feedback control, the red segment and the light pink segment, representing the respective parts of the red apple and the orange, are lost at frame number 45 due a segmentation instability: The red segment and the light pink segment merge and form a new segment, colored in light blue (see Figure 6A, middle panel). If feedback control is included, this segmentation instability is detected and the original segments can be recovered by increasing the temperature in steps of △*T* = 0.15. As a consequence, the segments can be continuously tracked, as shown in Figure 6A (lower panel). The segments representing the cup could be recovered using the same mechanism.

**Figure 7.**
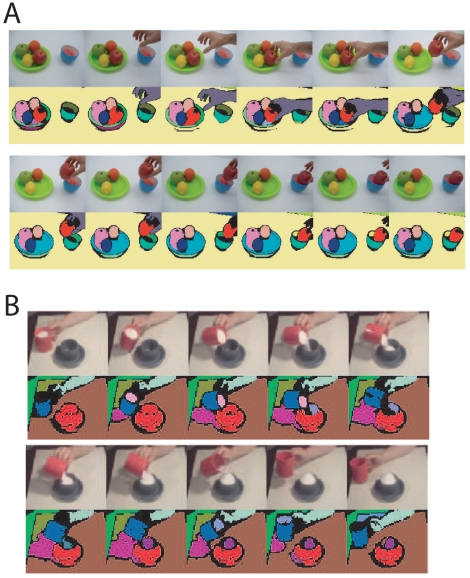
Segment tracking for real movies. **A** The algorithm (with feedback control) is applied to a movie showing a hand taking an apple from a plate (upper panel). The corresponding segment-tracking results are depicted below. **B** The results of the algorithm (with feedback control) for a movie showing the filling of a cup are shown. Segment-tracking results for other real movies can be found at http://www.nld.ds.mpg.de/∼eren/Movies.html/.

The work of the feedback controller is further illustrated in Figure 6B, where the segment size is plotted as a function of the frame number for the segments representing the red apple and the orange without and with feedback control, depicted as red, blue, brown and green lines, respectively. At frame number 45 the segment sizes of the red apple and the orange drop unexpectedly to zero (red and blue lines), thus indicating a segmentation instability (see Section 2.2). As a consequence, the feedback controller is activated and the temperature of the core algorithm is increased until the original segments are recovered (brown and green lines). The results for the whole movie are shown in Figure 7A.

We further applied the algorithm to another movie, showing the filling of a cup with sugar (Figure 7B). The movie is challenging because it contains light reflexions and changing shadows. However, the algorithm is capable of tracking the main segments of the movie, *i.e.*, the two cups and the hand.

We obtained similar segment-tracking results for other real movies, e.g., Moving Object, Making Sandwich, Opening a Book. Results can be found at http://www.nld.ds.mpg.de/∼eren/Movies.html.

**Figure 8.**
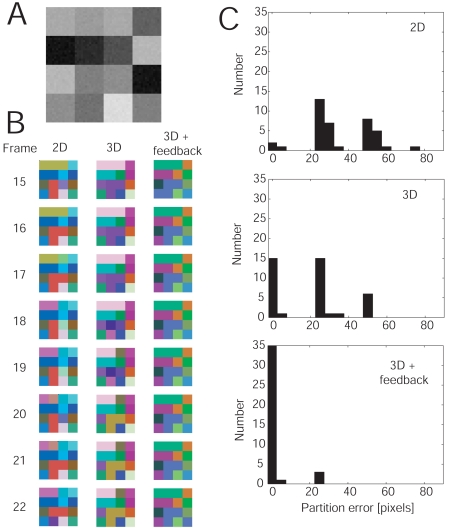
Improving partitioning consistency using 3D linking and feedback control. **A** From the original image consisting of 4 × 4 uniformly-valued squares, a static image sequence is created by adding Gaussian noise to each frame. **B** Segmentation results for our algorithm without 3D linking (no tracking), with 3D linking, and with both 3D linking and feedback control. **C** Respective histograms showing the number of frames as a function of the partition errors (see text).

We use an artificial image sequence to demonstrate that both 3D linking and feedback control improve consistency of the partitioning into segments of adjacent frames. The original image consists of 4 × 4 uniformly-valued squares. By adding Gaussian noise to the image, we create an image sequence of 40 frames (see Figure 8A). We apply the algorithm at *T* = 0.1 (i) without 3D linking and without feedback, (ii) with 3D linking and without feedback, and (iii) with 3D linking and with feedback. Example segmentation results are presented in Figure 8B. For case (i), the partitioning is often changing from frame to frame. The labels of adjacent frames only correspond to each other by chance, since no linking in 3D is employed (independent segmentations). For case (ii), the partitioning is more stable, but breaks still occur frequently. These breaks can be largely prevented by feedback control. We quantify the stability of adjacent image partitions by computing the spatial gradient for each segmented frame. We find a partition boundary if the gradient is larger than zero. The partition-boundary images of adjacent frames are then subtracted, the absolute value is taken, and the sum is computed over all pixels, which we call here the partition error. In Figure 8C, the histograms of the partition error over the whole sequence are shown for all three cases (i-iii) with mean partition errors (averaged over all frames) of 35.72, 19.44, and 2.59, respectively.

## 4. Discussion

We presented an algorithm for model-free segment tracking based on a novel, conjoint framework, combining local correspondences and image segmentation to synchronize the segmentation of adjacent images. The algorithm provides a partitioning of the image sequence in segments, such that points in a segment are more similar to each other than to points in another segment, and such that corresponding image points belong to the same segment. We tested the method on various synthetic and real image sequences, and showed stable and reliable results overall, thus fulfilling the most important requirement of segmentation algorithms. The method leads to the formation of stable region correspondences despite largely incomplete disparity or optic-flow maps. Similar algorithms for the extraction of region correspondences could potentially be constructed using other image segmentation algorithms, *i.e.*, methods based on agglomerative clustering [36, 37]. We decided to use physics-based model for its conceptual simplicity which allowed us to integrate local correspondence information in a straightforward way. It further has the advantage that the interacting parts are inherently converging to the equilibrium state and thus are not being trapped in local extrema (detailed balance). As a consequence, the result is independent of the initial conditions, allowing us to apply the algorithm to long image sequences via spin-states transfer. This allows for consistent segment tracking across many frames without additional assumptions, which is most of the time not immediately possible with other methods. In addition, no assumptions about the underlying data are required, e.g. the number of segments, leading to a model-free segmentation. This has the consequence that a single pixel of distinct gray value (compared to its neighbors) might define a single segment. In algorithms, which partition the image into a fixed and usually small number of segments, this phenomenon does not occur. This, however, is a problem as in all realistic situations one never knows how many segments exist and self-adjustment of the total number of segments is, thus, usually desired as compared to a pre-defined maximal number.

We further introduced a feedback controller which allows to detect segmentation instabilities, *i.e.*, merging and splitting of segments. The feedback controller adjusts the control parameter of the core algorithm in order to recover the original segments. This allows keeping track of segment even in long movies.

Segment tracking has been performed previously in the context of video segmentation [2–9]. Our method differs from these approaches in the choice of the segmentation algorithm, the way linking is achieved, and the addition of a feedback controller which detects segmentation instabilities. Superparamagnetic clustering allows a model-free unsupervised segmentation of the image sequences, including a self-adjustment of the total number of segments. Linking is introduced trough local correspondence information which synchronizes the spin-relaxation process of adjacent images. This approach has the advantage that the partitions of adjacent images are less prune to partitioning instabilities (see also Figure 8). Further, our method does not require corresponding regions to fulfill any segment similarity criterium. Finally, feedback control allows segmentation instabilities occurring in long sequences to be removed by assuming that “good” segments change their size in a continuous “predictable” manner.

There have been a few other approaches combining image segmentation with correspondence information. The work by Toshev *et al.* [38] uses a joint-image graph containing edges representing intra-image similarities and inter-image feature matches to compute matching regions. Joint segmentation has also been employed by Rother *et al.* [39] using histogram matching.

The controller employed in this model serves the detection and removal of segmentation instabilities. No assumptions about the objects giving rise to the measurements, *i.e.*, segments, are made here, except from that they cannot appear and disappear all of a sudden (object constancy) and are thus in some way predictable. Predictable behavior of objects provides the basis for tracking methods using optimal filters such as the Kalman filter [40], interacting multiple models [41], or particle filters [42]. In these cases however stronger assumptions about the nature of the objects are being made. Both some knowledge of the dynamics of objects (as then reflected by the measurements) and their appearance is assumed [43]. In the context of specific applications as for example the tracking of moving objects, Kalman-filter-based motion could potentially be combined with our method. Kumar *et al.* (2006) proposed a method for multitarget tracking in which blobs, *i.e.*, connected regions obtained from segmenting moving foreground objects via a background substraction method, are tracked with a Kalman filter while handling splits and merges between blobs. Certain elements of this approach may provide a means for further advancing the segment-tracking procedure described in this paper.

The method described here is related to energy minimization in Markov random fields which has been used to solve vision problems many times before [19, 29, 44–47] (see also Section 2.3). While the algorithmic procedures used to find the energy minimum share features with the ones employed by our method, fundamental differences exist between the methods. Superparamagnetic clustering aims at finding the equilibrium states of a Potts model without external field or data energy term at a certain temperature, and *not* a global energy minimum. Formulating vision problems in terms of energy minimization requires a data penalty term (or external field), necessitating some prior knowledge about the data that is being modeled. Hence, the solutions have to be considered less generic and quite different to ours by nature.

The algorithm has potential applications in model-free moving object detection and tracking by merging coherently moving segments (Gestalt law of common fate). The method is further applicable to action-recognition tasks, where certain characteristic action patterns are inferred from the spatiotemporal relationships of segments. First results for this problem are reported in [25]. In the future, texture cues may be incorporated into the algorithm to allow tracking of segments defined by texture.

Currently, the algorithm requires ≈ 4-5 s per frame for images of size 160 × 140 pixels and ≈ 43 s per frame for images of size 360 × 240 pixels (Taking-an-apple sequence) on an Intel Dual Core CPU with 3.16 GHz RAM (for each core). Since our goal is the development of a vision-front end for real-time video segment tracking on top of which other algorithms, *i.e.*, moving-object detection/tracking and action recognition, can be applied, we are currently investigating ways to improve computation time. Faster processing on a CPU could be achieved by improving the cluster identification step using a faster algorithm [48] and by applying the algorithm not to the pixels itself, but to atomic regions obtained from Canny edge detection followed by edge tracing and contour closing. The latter technique has been used before to improve speed in energy minimization [29]. Furthermore we are currently developing a parallel implementation on GPUs. So far, we reached frame rates between ≈ 10 – 23 frames/s for images of size 360 × 240 pixels (certain algorithmic procedures of the method have been replaced by alternative computation schemes to match the requirements of the parallel architecture).
